# c-MYC mediates the crosstalk between breast cancer cells and tumor microenvironment

**DOI:** 10.1186/s12964-023-01043-1

**Published:** 2023-01-31

**Authors:** Fang-yan Gao, Xin-tong Li, Kun Xu, Run-tian Wang, Xiao-xiang Guan

**Affiliations:** Department of Oncology, The First Affiliated Hospital of Nanjing Medical University, Nanjing, 210029 China

**Keywords:** c-MYC, Breast cancer, Tumor microenvironment, Crosstalk

## Abstract

The MYC oncogenic family is dysregulated in diverse tumors which is generally linked to the poor prognosis of tumors. The members in MYC family are transcription factors which are responsible for the regulation of various genes expression. Among them, c-MYC is closely related to the progression of tumors. Furthermore, c-MYC aberrations is tightly associated with the prevalence of breast cancer. Tumor microenvironment (TME) is composed of many different types of cellular and non-cellular factors, mainly including cancer-associated fibroblasts, tumor-associated macrophages, vascular endothelial cells, myeloid-derived suppressor cells and immune cells, all of which can affect the diagnosis, prognosis, and therapeutic efficacy of breast cancer. Importantly, the biological processes occurred in TME, such as angiogenesis, immune evasion, invasion, migration, and the recruition of stromal and tumor-infiltrating cells are under the modulation of c-MYC. These findings indicated that c-MYC serves as a critical regulator of TME. Here, we aimed to summarize and review the relevant research, thus to clarify c-MYC is a key mediator between breast cancer cells and TME.

**Video Abstract**

**Supplementary Information:**

The online version contains supplementary material available at 10.1186/s12964-023-01043-1.

## Introduction

*c-MYC* gene belongs to the *MYC* family, located on human chromosome 8, encodes the transcription factor c-MYC, which participates in cell cycle progression, proliferation, apoptosis, and cellular transformation [1, 2]. The expression levels of c-MYC are tightly modulated by several mechanisms involving transcriptional regulation of proximal promoter region [3]. As a transcription factor, c-MYC dimerizes with MAX (a helix-loop-helix leucine zipper protein), and then binds to DNA to regulate gene expression [4–6]. In breast cancer patients, c-MYC has been confirmed to be highly expressed and lead to the occurrence and development of breast cancer [7–10].

**Fig. 1:**
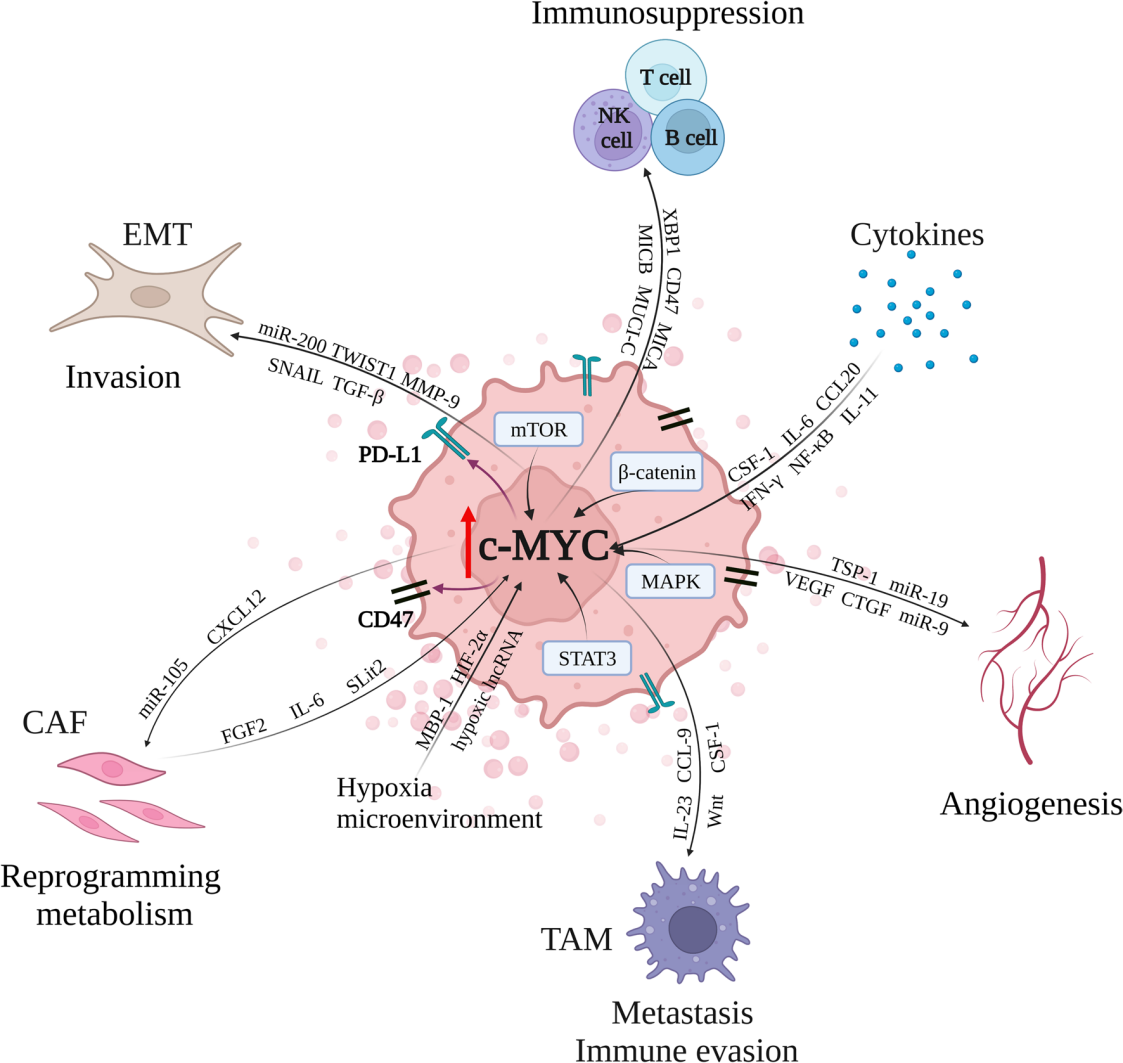
c-MYC is responsible for the crosstalk between breast cancer cells and tumor microenvironment. Within the tumor microenvironment, c-MYC can be regulated by several signaling proteins, such as STAT3, MAPK, β-catenin and mTOR. In addition, c-MYC contributes to the regulation of angiogenesis, the function of CAF and EMT. Moreover, c-MYC is able to modulate TAM, NK cell, T and B cell, as well as the expression of PD-L1 and CD47, thus leading to the immune evasion and immunosuppression. Besides, the tumor microenvironment can affect c-MYC expression in turn by various cytokines, the hypoxia microenvironment and the factors released from CAF

Accumulating evidence revealed that c-MYC is a critical regulator of the tumor microenvironment (TME), and involved in the stromal cell growth and angiogenesis (Fig. 1) [11–13]. TME contains many different types of cells, including cancer-associated fibroblasts (CAFs), tumor-associated macrophages (TAMs), vascular endothelial cells (VECs), myeloid-derived suppressor cells (MDSCs), immune cells, inflammatory cells, adipocytes, and myoepithelial cells [14, 15]. Non-cellular components are also important parts of TME, such as extracellular matrix (ECM), extracellular vesicles (EVs), soluble cytokines and signaling molecules [16–18]. Moreover, the blood vessels and lymphatic system are also included in TME [19].

In breast cancer, increasing studies have revealed the close relationship between TME and tumor progression. This might be dependent on the c-MYC mediated crosstalk between TME and breast cancer cells [20–23]. Considering the vital role of c-MYC in both breast cancer cells and TME, this review aimed to summarize the functions and underlying mechanisms of c-MYC acts in the communication between breast cancer cells and TME.

## c-MYC coordinates the crosstalk between breast cancer cells and angiogenesis

Like most solid tumors, new blood vessels are required if breast cancer cells are to grow beyond a few millimeters in diameter [24]. New blood vessels provide nutrients to tumor cells, allowing cells to proliferate and spread to other locations. Vascular endothelial growth factor (VEGF) is the major mediator of angiogenesis and functions by binding to its receptors on vascular endothelial cells. The oncogene expression, different growth factors, and hypoxia can induce the expression of VEGF [25–27]. Except for the stroma in the TME stimulates endothelial cell proliferation, the secret of VEGF from tumor cells can promote the formation of new blood vessels [28]. In breast cancer cells, targeting c-MYC has been demonstrated to inhibit tumor angiogenesis [29–31]. Mezquita et al. [32] found that VEGF expression was increased by c-MYC, which could stimulate the mRNA translation of VEGF. In addition, c-MYC can promote VEGF expression alone or in combination with Erα on the VEGF promoter [33]. By increasing the expression of miR-9, c-MYC caused the downregulation of E-cadherin and activation of β-catenin signaling, thus inducing the upregulation of VEGF and the tumor angiogenesis [34]. Pro-angiogenic miR-19 is also modulated by c-MYC, which directly targets anti-angiogenic proteins CTGF and TSP-1, thereby promoting angiogenesis [35]. Notably, c-MYC is able to affect angiogenesis by regulating VEGF, while VEGF can in turn affect c-MYC expression. As reported before, VEGF raised the expression level of c-MYC by activating STAT3 and enhances the self-renewal of breast cancer stem cells [36]. Except for inhibiting VEGF expression, c-MYC also decreased TSP-1 expression, facilitating tumor neovascularization [37, 38]. Combined with above, c-MYC can promote angiogenesis by affecting TME components such as VEGF and TSP-1 in breast cancer. Therefore, the expression of c-MYC can conversely modulated by VEGF in breast cancer.

## c-MYC mediates the function of CAFs on breast cancer tumor growth

In TME, CAFs is the most common stromal cells, which deposits and modifies ECM. CAFs is generated due to the activation of normal fibroblasts under stimuli such as inflammatory signals or contact signals [39]. As previous studies reported, CAFs secrets soluble factors or modulates ECM to simulate tumor progression [18, 40–42]. Notably, c-MYC in breast cancer cells can promote tumor progression through CAFs. Yan et al. discovered that c-MYC increases the expression level of miR-105 in tumor cells vesicles. After receiving vesicles, c-MYC is upregulated in CAFs, inducing the reprogramming metabolism to promote tumor growth [43]. Besides, in mammary epithelial cells, c-MYC impact fibroblasts significantly. Mammary epithelial cells with c-MYC overexpression secrete IGF-I and IGF-II, which could subsequently induce fibroblasts via IGF-1R [44]. Valverius et al. [45] showed that the co-culture of human mammary epithelial cells overexpressing c-MYC with mammary fibroblasts induces both anchorage-independent and anchorage-dependent proliferation.

Moreover, the secretion of CAFs contributes to the expression of c-MYC in breast cancer cells, thereby promoting tumor growth. For instance, the medium of CAF induces cyclin D1, c-MYC, MMP-2 and MMP-9 expression in breast cancer cells, accelerating proliferation, migration and invasion [46, 47]. CAFs produce fibroblast growth factor 2 (FGF2), followed up with activation and recruition of ERα and PRBΔ4 to the MYC regulatory sequence leading to the breast cancer growth [48]. In breast cancer cells, TCF12 stimulates the activation of c-MYC/Cyclin D1 pathway to accelerate cancer growth via facilitate CXCL12 of CAFs [49]. Besides, c-MYC is required to maintain the cell growth of CAFs. Knockdown of c-MYC in CAFs reduced cyclin D1, cyclin E and E2F1 expression whereas increases p21^clip1^ expression, resulting in impaired proliferation of CAFs [50].

In addition to the promoting effect of CAFs on breast cancer, some studies have also shown that CAFs slow down breast cancer growth by downregulating c-MYC. Slit2, produced by stromal fibroblasts, can bind to the Robo1 receptor to inhibit carcinogenesis through blocking the nuclear translocation of β-catenin by modulating the PI3K/AKT axis and downregulating c-MYC [51]. In ERα-negative breast cancer cells, CAF-secreted IL-6 decreases c-MYC expression and suppresses tumor growth [52]. Starved fibroblast supernatants reduced MYC expression levels in breast cancer cells and induced cancer stem cells death [53]. Combined with above, CAFs have a dual function on breast cancer tumor growth and c-MYC is a critical mediator during the process.

## c-MYC regulates the immune response in TME

Immune cells in TME mainly contains TAMs, dendritic cells, MDSCs, T cells, B cells, natural killer (NK) cells, neutrophils and others, which are tightly associated with the immune response [17, 54, 55]. Many researches have shown there is a close relationship between c-MYC and immune cells in TME.

### c-MYC is elevated in TAMs

Macrophages are always divided into two groups according to their polarization and activation markers: M1 and M2. The M1 macrophage speeds up the inflammatory response, whereas the M2 type slows it down. Monocytes eventually develop into TAM after being enriched to tumor tissue via numerous signals in the tumor microenvironment [56, 57]. TAMs are considered M2-like macrophages because they suppress local immunity and support tumor growth. c-MYC is elevated in TAM and is involved in suppressing immunity, facilitating breast cancer tumor growth, and modulating the expression of proto-oncogenes (VEGF, MMP9, HIF-1α, TGF-β, MRC1, ALOX15) [58–60]. In TAM, Wnt ligands released from tumor cells can activate the Wnt/β-catenin axis, thereby activating c-MYC, leading to M2 polarization of TAM, which leads to tumor development, migration, and metastasis [61]. Liu and colleagues also found that c-MYC mRNA was significantly upregulated in macrophages after CSF-1 stimulation, driving macrophage proliferation. In addition, TAM infiltration is involved in the induction of c-MYC expression via IL-6/STAT3 pathway, indicating that there is a potential feedback loop mechanism to regulate the expression of c-MYC in TME [62]. Moreover, pro-inflammatory, stimulated by LPS and IFN-γ, inhibits the expression of c-MYC and the proliferation of macrophages [63]. Breast cancer cells with high c-MYC expression are more prevalent, promoting tumor progression through macrophages. By modulating SRC-1 expression, c-MYC activates colony-stimulating factor 1 (CSF-1) and enriches macrophages to enhance breast cancer metastasis [64]. The fusion of macrophages and breast cancer cells strengthened the activity TCF/LEF transcription factor and promoted the expression of downstream target genes (including cyclin D1 and c-MYC), leading to the promotion of tumor progression, metastasis, and EMT process [65]. Therefore, the intercommunication between breast cancer cells and TAM upregulates c-MYC expression, accelerating the breast cancer progression.

### c-MYC inhibits tumor immune response

Tumor infiltrating lymphocytes (TILs) mainly clarified as T lymphocytes, B lymphocytes, and NK cells. In breast cancer, TIL acts a crucial role in mediating the response to chemotherapy [66]. Patients can benefit from treatment with increased TIL. TILs can be activated under the stimulation of various factors and participate in the immune response of tumors. Upon the activation of c-MYC, there is an immediate exclusion of T, B and NK cells within TME which are mainly caused by the up-regulation of chemokine CCL9 and IL-23 [67–69]. Interestingly, a study reported by Han and colleagues found that with c-MYC inhibitors, the percentage of CD3+ cells and natural killer cells was elevated addition whereas regulatory T cells were on the decline [70]. In triple-negative breast cancer (TNBC) cells, MUCI-C can recruit c-MYC to the promoter region of PD-L1, promoting PD-L1 expression. Inhibition of MUCI-C also suppressed c-MYC expression, resulting in an increase in IFN-γ in the CD8+ T cell population [71]. Upregulation of IFN-γ in CD8+ T cells has been demonstrated to aid tumor shrinking in studies [72]. CD47 is commonly upregulated in multiple tumors [73]. CD47 interacts with SIRPα, and initiates an inhibitory signaling pathway which causes tumor cells to evade phagocytosis by macrophages. PD-L1 is expressed on the surface of tumor cells to deliver inhibitory signals after interacting with ligands. It has been revealed that c-MYC could promote CD47 and PD-L1 expression by binding to the promoters of CD47 and PD-L1 genes to suppress antitumor immune responses [74]. As an immune checkpoint protein, PD-L1 on tumor cells can lead to the evasion of immune response, and then accelerate tumor progression. A variety of oncogenes, such as MAPK and AKT/mTOR, have been illustrated to regulate the expression of immune checkpoint [75, 76]. A possible mechanism is that these carcinogenic pathways are able to regulate c-MYC expression, which may be how they regulate the expression of immune checkpoints, such as PD-L1 [77]. Therefore, c-MYC may be a critical mediator between these carcinogenic signaling pathways and tumor immune response. In TNBC, c-MYC attenuates STING-dependent innate immunity by inhibiting the activation of dendritic cells and T cells. Breast cancers with c-MYC knockdown contain more CD3+ and CD8+ T cells, and c-MYC impairs T cell infiltration in vivo, leading to the formation of a “non-inflammatory tumor” microenvironment that induces immune evasion [78]. Furthermore, c-MYC inhibits antitumor immune responses by downregulating MICA and MICB via miR-17, resulting in reduced cell lysis in breast cancer cells [79]. In addition, XBP1 promotes NK cell proliferation in part by directly transactivating c-MYC expression [80].

In summary, c-MYC promotes the tumor progression of breast cancer by inhibiting the activation of immune cells in the TME.

## Cytokines promotes the expression of c-MYC

Cytokines play an important role in creating an inflammatory TME and participate in the occurrence and development of tumors [81]. They are released by numerous cells, such as immunological cells, endothelial cells, and epidermal cells. Cytokines, most of which are small molecule polypeptides, exert their functions by interacting with receptors on the surface of cell membranes. Vast majority of cytokines contribute to tumor progression. In breast cancer cells, Sapi and colleagues demonstrated that cytokine CSF-1 induces expression of c-MYC, enhancing tumorigenicity as well as invasive potential [82]. Besides, c-MYC mRNA was significantly upregulated in macrophages after CSF-1 stimulation, boosting macrophage proliferation [63].Via JAK1/STAT3 signaling pathway, IL-11 induces c-MYC expression to promote breast cancer bone metastasis [83]. The chemokine CCL20 activates AKT pathways to enhance transcription of c-MYC, which is responsible for breast cell proliferation [84]. However, IFN-γ causes a decrease in c-MYC in normal human mammary epithelial cells and inhibits proliferation [85]. In addition, IL-6, VEGF and NF-κB promotes the expression of c-MYC via STAT3, leading to the development of breast cancer invasion and metastasis [86, 87]. Another study showed that breast cancer cells cultured with cytokines could activate Src, which promoted the upregulation of SOX2 and c-MYC. Furthermore, SOX2 stimulated c-MYC expression and enhanced cancer stem-like properties [88]. Taken together, multiple cytokines can promote breast cancer progression by upregulating c-MYC expression in cancer cells.

## Hypoxic TME induced c-MYC expression

Hypoxia is a vital feature of the TME, which is mainly due to the abnormal function of new blood vessels. Meanwhile, the rapid growth of tumor cells demands enough oxygen, exacerbating hypoxia. Many literatures report that hypoxia contribute to the rapid development of breast cancer [89]. Hypoxic TME has been reported to enhance HIF-2α expression, resulting the overexpression of c-MYC. This increases the stemness of breast cancer [90]. MBP-1 was reported to regulates the expression of c-MYC negatively. Hypoxia can block the interaction of MBP-1 with the c-MYC promoter, thus fades the negative regulation of MBP-1 on c-MYC. Furthermore, high expressed c-MYC stimulates aerobic glycolysis, reinforcing the adaptation of tumor cells to oxidative stress [91]. Hypoxia can also cause high levels of a novel hypoxic lncRNA KB-1980E6.3 expression. KB-1980E6.3 could elevate c-MYC protein level by maintaining the mRNA stability of c-MYC and is responsible for the breast cancer stem cells (BCSCs) self-renewal and stemness maintenance [92]. Under hypoxia-reoxygenation conditions, the increase of c-MYC decreases the tumor suppressor NDRG1 expression. Based on this, hypoxia-reoxygenation enhances the metastasis of breast cancer [93].

## Conclusions and perspectives

As a crucial oncogenic transcription factor, c-MYC regulates numerous genes and factors in breast cancer cells and TME. Notably, the changes of TME are responsible for the progression of breast cancer. For breast cancer patients, TME is therefore a promising therapeutic target. In recent decades, some targeted therapies have been developed for breast cancer patients, however, the strategies are commonly focused on tumor cells themselves [94, 95]. The genome of breast cancer cells is yet unstable, and gene mutations very probably occur after targeted therapy. This may cause the occurrence and development of drug resistance. Compared to breast cancer cells, cells exist in TME are relatively stable on the genome. Therefore, the probability of cell genes mutation might be reduced significantly, as well as the drug resistance, if the therapy is targeted at the tumor microenvironment. The characterization of TME in breast cancer has uncovered the crosstalk between the diverse molecules involved, including c-MYC. c-MYC serves as a key mediator to closely connect the TME and breast cancer cells by regulating the diverse factors in TME. Interestingly, the expression of c-MYC can be influenced by the components of TME in turn, indicating c-MYC and TME are tightly related in breast cancer. Therefore, c-MYC possesses great potential to be an effective target for breast cancer by interfering the balance and blocking the crosstalk between TME and tumor cells. However, which factor is relatively more important during the regulation between c-MYC and TME in breast cancer, the angiogenesis? immune cells? or CAF? In addition, it has been reported in a previous study that nanomedicines containing c-MYC targeted inhibitors in M2 macrophages, but have less influence on M1 macrophages that kill tumor cells [60]. This suggests to us that with the advancement of technology, we can accurately target c-MYC in some specific cells, which contribute to the oncogenic process during the communication between breast cancer cells and TME.

Although c-MYC is a potential therapeutic target for cancers, specific molecules targeting c-MYC are hardly to construct, because of the largely intrinsically disordered structure, lacking catalytic activity, and its nuclear localization [96]. Nonetheless, a few strategies for targeting c-MYC have been developed [96, 97]. These strategies are mainly divided into direct and indirect treatment. Among them, the direct treatment is the therapy directly targeting c-MYC, such as OmoMYC [98]. OmoMYC is a 90-amino acid peptide that imitates the bHLHLZ domain of c-MYC which can antagonize c-MYC and attenuate cancer cell growth [99–101]. It was demonstrated that the intratumor injection of OmoMYC suppressed tumor growth via reducing PD-L1 in a TNBC model [102]. Dependent on its favorable efficacy and low toxicity, OmoMYC is being evaluated for clinical treatment. For small molecule inhibitors, KJ-Pyr-9 [103], Mycro3 [104], MYCMI-6 [105] and MYCi975 [70] are the promising low molecular weight c-MYC antagonists. Among them, KJ-Pyr-9 showed high binding affinity with c-MYC, and has been revealed to inhibit TNBC tumor growth without side toxicity [103]. Apart from above, some other potential strategies for targeting c-MYC have also been explored, such as inhibiting the translation of c-MYC [106], and breaking the interaction between c-MYC and binding proteins [107]. Currently, although none molecules targeting c-MYC has been approved in clinical application, increasing studies have focused on resolving the limitations mentioned above to develop feasible approaches for the treatment of tumors. Recently, as the first direct inhibitor of c-MYC to pass human first clinical test, OMO-103 has shown anti-tumor activity and safety in patients with advanced solid cancers according to first-in-human data presented at the EORTC-NCI-AACR Symposium on Molecular Targets and Cancer Therapeutics, shedding light on the clinical treatment by targeting c-MYC [108]. In breast cancer patients, the alternative MYC and chromosome 8 copy number anomalies might characterize the responsive or nonresponsive subgroups of HER2+ tumors to trastuzumab [109]. Although some clinical trials involving c-MYC in breast cancer have been reported, the clinical trials of drugs direct targeting c-MYC in it are lacking [98]. Therefore, more studies focused on targeting c-MYC are needed for developing novel therapeutic strategies for breast cancer patients.

Collectively, c-MYC mediates the crosstalk between breast cancer cells and TME to regulate tumor progression. Nevertheless, more study is urgent for further characterizing more details about c-MYC in the mediation of TME and breast cancer cells, which will be beneficial for developing better and accurate therapeutic protocols for the management of breast cancer.

## Data Availability

Not applicable.

## Abbreviations

TME: Tumor microenvironment
CAFs: Cancer-associated fibroblasts
TAMs: Tumor-associated macrophages
VECs: Vascular endothelial cells
MDSCs: Myeloid-derived suppressor cells
ECM: Extracellular matrix
EMT: Epithelial–mesenchymal transition
EVs: Extracellular vesicles
VEGF: Vascular endothelial growth factor
FGF2: Fibroblast growth factor 2
NK: Natural killer
CSF-1: Colony-stimulating factor 1
TILs: Tumor infiltrating lymphocytes
TNBC: Triple-negative breast cancer
BCSCs: Breast cancer stem cells
